# Book Reviews

**Published:** 1897-02

**Authors:** 


### Book Reviews.

MEDICAL JURISPRUDENCE, FORENSIC MEDICINE AND TOXICOLOGY. By R. A. WITTHAUS, A. M., M. D., and TRACY C. BECKER, A. B., LL.B., and a staff of collaborators. In four royal octavo volumes. Volume IV., Toxicology. New York: William Wood & Company. 1896.

The fourth and final volume of this work is devoted to the exclusive consideration of toxicology and is by one of the editors, Prof. R. A. Witthaus, M. D., whose writings and teachings are well known to the medical and legal professions. He has often been summoned as an expert witness in important trials before the courts, and has frequently succeeded in solving most difficult problems in forensic medicine.

This treatise begins with a historical introduction, which possesses great interest for the student, and which is accompanied with an extensive list of bibliographic references. General toxicology is then taken up and is considered under various heads in the next 145 pages. Witthaus is most interesting in this division, and especially under the section, absorption of poisons, in which we find much of value to every physician who would understand the methods of action of corrosives and poisons, as well as methods of the elimination of poisons. A knowledge of the proper treatment of poisoning should be the privilege or duty of every physician in active practice, and in this section will be found a vast storehouse of information on that subject. All, too, who are likely to be called into the courts must study carefully the evidence in cases of murder by poisoning, and the duties of physicians in cases of poisoning. There are several other important subdivisions that are considered in this section and which, as a whole, we deem the most exhaustive that has ever been published.

Special toxicology is now taken up and occupies the next 100 pages. This section includes dissertations on corrosives, such as mineral acids, fixed mineral alkalies, ammonium hydroxide and carbonate, and the halogens, chlorin, bromin and iodin. Witthaus states that the halogens are four elements—namely, fluorin, bromin, chlorin and iodin,—that exist in compounds more or less allied to or resembling sea salt, but that fluorin, although probably one of the most corrosive, has no practical toxicological interest, owing to exceptional conditions necessary for its liberation. The subdivision, iodin, furnishes interesting reading, even for those who are not specially attracted to the general subject of toxicology. It is here stated that the so-called colorless tincture of iodin contains no free iodin, but triethylamin, ethyl, iodide and ammonium iodide, along with alcohol and some free ammonia. It will thus be seen that this preparation is next to valueless as an external application.

Mineral poisons next engage attention and occupy the following 300 pages of the book. We are impressed all through this section with the careful and painstaking character of the author's work, but find it inexpedient to discourse upon it at length. If a physician desires to obtain an exhaustive dissertation upon any one of the mineral poisons, he will find it here embodied and most learnedly set forth. Especially do we commend the subdivision relating to arsenic.

Coming now to the vegetable poisons, 234 pages are here absorbed in their treatment. Here again we find the same exhaustive, painstaking application made in discoursing upon the conditions that frequently engage the practising physician, sometimes as the result of innocent overdosing in the vain attempt to urge therapeutics to a limit beyond their resources ; or again from idiosyncrasy toxic results ensue from medicinal doses, all of which is most masterfully considered in this section. Especially do we find absorbing interest in what Witthaus says regarding opium and its preparations. Taken altogether perhaps opium poisoning is more common than any other and should be studied with care by all physicians. There is no better setting forth of its various phases than in this work.

Animal poisons, such as poisonous foods, occupy attention in the next ten pages ; and synthetic poisons are also considered in the following ten pages, which conclude the treatise.

Then follows a table of cases referred to in the volume and finally an elaborate index. This volume is a fitting conclusion to a great work that will always stand supreme in the literature of medical jurisprudence. It is a treatise that will become renowned for its classic research and for its exhaustive treatment of the subjects which it considers.

---

A Treatise on Obstetrics. For students and practitioners. By Edward P. Davis, A. M., M. D., Professor of Obstetrics and Diseases of Infancy in the Philadelphia Polyclinic ; Clinical Professor of Obstetrics in the Jefferson Medical College of Philadelphia. In one octavo volume of about 600 pages, with 217 engravings and thirty full-page plates in colors and monochrome. Cloth, $5.00 ; leather, $6.00. Philadelphia and New York : Lea Brothers & Co., Publishers. 1896.

If another work were needed on obstetrics at this time we know of no one more competent to prepare a practical treatise than this author. The favorable reception given his obstetric manual indicates that a more complete and elaborate treatise will be received by the profession with favor.

After a short introduction the author presents the subject of pregnancy and labor, to which he devotes thirteen chapters. In the first one, obstetric diagnosis is considered ; in the second, differential diagnosis of pregnancy ; in the third, diagnosis of advanced pregnancy. We like this classification of obstetric diagnosis, and find in the text much that may be regarded as belonging to newer methods. Many questions relating to this part of the subject are exceedingly trying to the beginner and he should have definite and explicit teaching in regard to the differential diagnosis in early as well as late pregnancy. In both states questions of great importance arise, but of widely different natures.

The origin and growth of the ovum and the development of the germ is considered in the next chapter; and in chapter five the growth and development of the embryo is presented. Davis explains these intricate subjects with a rare clearness that will be of advantage to students. The physiology of pregnancy next occupies attention; and in chapter seven follows a consideration of the pathology of pregnancy. This, as might be expected, occupies considerable space, as it is one of the most important subdivisions of this section. If we had time and space to analyse this chapter it might prove of exceeding interest, but far better will it be for obstetric physicians to read the author's original text. The toxemia of pregnancy is well considered both as to its prevention and cure, while minute directions are given for examining the urine with reference to the presence of toxins and the amount of urea eliminated.

Normal labor and its management is taken up in chapter eight, which is unusually interesting in its condensed details. Since by far the larger proportion of labors are normal, it is essential that a full understanding of the natural process should be well understood in order that departures from the health line may be sharply drawn and promptly recognised. Coming immediately after this subject, the author presents for our consideration abnormal labor in chapter nine, the cranium presenting; in chapter ten, the face presenting and its management; in chapter eleven, the breech presenting. The management of head-last labors is one of the most interesting fields in obstetric practice, for in it is afforded an opportunity, generally speaking, for the display of obstetric skill and judgment. When the obstetric physician has successfully dealt with the after-coming head and saved two lives without impairment of future health to either, he has accomplished a professional feat that entitles him to credit.

Labor resulting in the impaction of the fetus, or what may be termed impossible labor, is briefly considered in chapter twelve, and in our view has not been accorded as much elaboration as is consistent with the subject. The complication of pregnancy with fibroids is not an uncommon condition and sometimes requires the most skilful application of obstetric science. This section of the book closes with chapter thirteen, which is devoted to the consideration of multiple pregnancy.

In the next section the pathology of labor is taken up and the first chapter presents the subject of its induction. This is a branch of the obstetric art that requires peculiar skill and judgment to conduct in the proper manner, since the methods that are applicable to one condition may not be equally safe and advantageous in another; in other words, the deliberate induction of labor in a deformed pelvis previously planned for by consultation is one thing, and the induction of labor as a sudden immediate expedient, made necessary by unheralded eclampsia, is quite another.

Abnormal labor pains furnish the subject for the second chapter; hemorrhage, including placenta previa, the third; eclampsia, the fourth; sudden death during labor, the fifth; labor complicated by disproportion between the pelvis and fetus, the sixth; labor in large pelves, the seventh; in contracted pelves, the eighth; in deformed pelves, the ninth; in cases complicated by tumors, the tenth; and finally septic infection is discoursed upon in the last chapter, the eleventh of this section.

Obstetrical operations form the subject of section three, to which the usual consideration is given. Beginning with episiotomy and the suture of perineal rents, next comes the consideration of the forceps, and then follow in their order version and extraction, symphyseotomy, Cesarean section, Porro's operation, embryotomy, and obstetric curettement, or operative emptying of the uterus.

The next section, four, is devoted to the consideration of abortion, extrauterine pregnancy, and the puerperal state. Under this head are included the repair of laceration of the cervix caused by labor, repairs of injuries to the pelvic floor and the perineum; insanity and nervous disorders accompanying the puerperal state and lactation.

Sections five and six relate to the infant. Infancy in health and disease forms the subject of section five, and the diseases of infancy that of section six. The final section of the book, seven, is devoted to the consideration of the jurisprudence of obstetrics. This last is really an interesting department, one frequently omitted in obstetric treatises, but one upon which every obstetric physician should have ample information.

A well-prepared index closes this valuable treatise. We believe it will take rank among the best, and is more than likely to be a popular text-book with students. The illustrations are numerous, largely original and many of them of admitted excellence.

---

A Manual of Obstetrics. By W. A. Newman Dorland, A. M., M. D., Assistant Demonstrator of Obstetrics, University of Pennsylvania; Instructor in Gynecology in the Philadelphia Polyclinic, etc. Octavo, pp. 760. Illustrated. Philadelphia: W. B. Saunders, 925 Walnut street. 1896.

At the first glance at the title of this book one is led to inquire, Is there a necessity for another obstetric manual? Judging by the number already at hand and especially of the popularity of King's that has so long held sway, the answer is tempted to be in the negative. But if one cares to pursue the subject further, and examine into the special plan of this work, he will be led to lend approbation to its existence.

It is constructed on the basis of a combined clinical, physiologic and pathologic work, which is a rational way in which to present the subject. While it is an agreeable fact to contemplate that most labors are physiologic, yet there is on the other hand as civilisation advances an apparent increase of pathologic pregnancies and deliveries. It behooves a man who offers his service as an obstetrician at this time to master the whole subject from pathologic, physiologic and clinical standpoints, and to be able to detect early that kind of a labor in which scientific aid will be required.

If pathologic labors are diagnosticated sufficiently early most of them could in general be led to a happy conclusion. The old dogma of meddlesome midwifery is not satisfactory to the modern obstetrician. If, however, he is called upon to act, he must have a reason for his action, and not be groping in the dark as to what his duty may be in a given case.

Young men will derive much benefit from the study of this manual, and will in it find many aids to a higher cultivation of the obstetric art, as well as a stimulus to acquire a better understanding of the responsibilities attaching to its practice.

The style of the book is that of the uniform edition of Saunders' new aid series and it is well printed and admirably illustrated.

---

Minor Surgery and Bandaging. By Henry R. Wharton, M. D., Demonstrator of Surgery in the University of Pennsylvania. New (third) edition. In one 12mo volume of 594 pages, with 475 engravings. Philadelphia: Lea Brothers & Co. 1896.

Two editions of this excellent work have already been commented upon in the Journal. The first was put forth in 1891, and within the brief period of five years the third now presents itself. We expressed ourselves in decided terms as to the value of this little work on both former occasions, stating that it was in our judgment one of the best books of its kind extant. It has been thoroughly revised in this edition, bringing it forward to the present period. It should be borne in mind that the text covers considerably more than its title would indicate; that is to say, in addition to ample details concerning minor surgery it gives valuable instructions regarding fractures, amputations, resections, trephining, operations on nerves and tendons, tracheotomy, laryngotomy, intubation, operations on the kidney and colon, lithotomy and osteotomy.

But most important of all in our view is the section on bandaging. Here Wharton has distinguished himself beyond any other author, whether in regard to text or illustrations. While the text itself is clear, the illustrations are so perfect, most of which are half-tone reproductions, that it is difficult for the novice to be misled in regard to the application of any sort of bandage to any part of the body for any purpose known in the domain of surgery.

The first part of the work comprising 108 pages is devoted to this subject, and if it stopped here it is well worth the price asked by the publishers, but special bandaging instructions are again given under appropriate heads, as fractures, dislocations and injuries. We repeat that Wharton has done the profession of medicine incalculable service, especially students and junior members of the profession, in preparing this work, and we have no doubt that it will continue to be appreciated, and that this edition will be exhausted quite as soon as the others have been. It, too, is one of the handsomest made-up books that has come to our table in many a day.

---

A HANDBOOK OF PATHOLOGICAL ANATOMY AND HISTOLOGY. With an introductory section on Post Mortem Examinations and the Methods of Preserving and Examining Diseased Tissues. By FRANCIS DELAFIELD, M. D., LL. D., Professor of the Practice of Medicine, College of Physicians and Surgeons, Columbia College, New York, and T. MITCHELL PRUDDEN, M. D., Professor of Pathology and Director of the Laboratories of Histology, Pathology and Bacteriology, College of Physicians and Surgeons, Columbia College, New York. Fifth edition. Octavo, pp. viii.—846. Illustrated by 365 wood-engravings, printed in black and colors. New York: William Wood & Co. 1896.

The fifth edition of this standard work will be welcomed by students of pathology and histology throughout the universe. We have heretofore in these columns expressed our opinion in considerable detail of the value of the treatise, and a perusal of this edition does not induce us to modify those opinions except to further accentuate them and to increase, if it were possible, their cogent force.

In this work may be obtained that important knowledge essential for the making of autopsies, the preservation of tissues as well as their preparation for microscopic study, and the outlines of methods of study of pathologic microörganisms. The authors, too, have described concisely the lesions of infectious diseases, including, as far as known, the microörganisms concerned in their causation, as well as the various phases of degeneration and inflammation, the character of tumors, the special lesions of different parts of the body, of general diseases, of poisoning, and of violent deaths. These, too, have been illustrated whenever or wherever pictures could be made available to elucidate the text.

In this edition all sections of the book have been revised, and some of them rewritten to a considerable extent, in order that they may embrace recent contributions to pathological and histological science. Many new engravings also have been added and the section on the blood has especially received elaborate attention at the hands of Dr. James Ewing. This work has been a leading textbook in most of our medical colleges since it first appeared, and it is so familiar to physicians and students that we need only accentuate the fact that the fifth edition is the most satisfactory general treatise on the subjects with which it deals that we have knowledge of, and it will of necessity command a constantly increasing sale.

Twentieth Century Practice. An International Encyclopedia of Modern Medical Science. By leading authorities of Europe and America. Edited by Thomas L. Stedman, M. D., New York City. In twenty volumes. Volume VII., Diseases of the Respiratory Organs and Blood, and Functional Sexual Disorders. New York: William Wood & Company. 1896.

The contributors to this volume are Charles W. Allen, of New York, who writes on functional disorders of the male sexual organs; Jules Comby, Paris, who discourses upon rachitis; Charles Green Cumston and Ernest W. Cushing, of Boston, who have prepared a section on disorders of menstruation; James M. French, Cincinnati, who tells us of the chemical and microscopical examination of the urine; E. Fletcher Ingals, Chicago, who has prepared the section on hay fever; E. Main, Paris, who is the author of the dissertation on diseases of the diaphragm; Franz Riegel, Giessen, who writes on asthma, and Herbert B. Whitney, Denver, who discourses exhaustively on diseases of the pleura. The latter subject, comprising 126 pages, opens the volume and is followed by asthma, 55 pages, hay fever, 25 pages, diseases of the mediastinum, 12 pages, diseases of the diaphragm, 14 pages, diseases of the blood, 300 pages, rachitis, 35 pages, disorders of menstruation, 30 pages, functional disorders of the male sexual organs, 58 pages, chemical and microscopical examination of the urine, 120 pages.

The usual excellent index closes the volume, which makes the series complete up to and including Volume VIII., which latter was issued out of its regular order; the next volume will therefore be Volume IX. We have heretofore expressed an opinion of the character of this massive treatise and need not here repeat it. The reputation of the authors of the several monographs is a sufficient guaranty of their excellence. Whoever desires to have the latest literature for reference in his library will be sure to subscribe for this comprehensive encyclopedic group of books.

---

A Manual of Clinical Diagnosis by Microscopical and Chemical Methods. For students, hospital physicians and practitioners. By Charles E. Simon, M. D., late Assistant Resident Physician Johns Hopkins Hospital, Baltimore. In one octavo volume of 504 pages, with 132 engravings and ten full-page colored plates. Cloth, $3.50. Philadelphia and New York: Lea Brothers & Co. 1896.

This treatise enters a new field of authorship; not but that the importance of clinical chemistry and microscopy have been well known, but it is a curious fact that they have been either imperfectly or illy applied. The physicians in general practice have considered themselves either too busy or too indolent to make use of the methods treated of in this book, but it is now quite time that a change were made in regard to the diagnosis and treatment of disease. It is essentially necessary that a physician should have a laboratory where he may make microscopic and chemic analysis of the fluids and tissues of the body, and if he cannot attend to the matter himself he should employ a laboratory assistant for that purpose. Accurate diagnosis leads to exacter, better and more successful methods of treatment.

Simon clearly points out the importance of chemic and microscopic methods in diagnosis, and he tells how to apply them. The plan of his work is to devote a single chapter to each fluid or class of fluids. For instance, the blood takes up the first chapter; secretions of the mouth the second, gastric juice and stomach contents the third, the feces the fourth, sputum the sixth, the urine the seventh, and so on to the end. Some of the chapters are exhaustive, especially those relating to the blood and the urine. On the other hand the nasal secretion is dismissed with a single page, which will appear almost scandalous to the average laryngologist.

The book all through protrays an experience with the subject, and no doubt the author has given the most consideration to those branches of it which he deems the most important. The illustrations are especially to be commended for their excellence, some of which are quite new and are in colors. The appendix is an ample key to the text and the book taken all together is one of the most valuable contributions to the literature of medicine in 1896.

---

A Text-Book of Diseases of the Nose and Throat. By Francke Huntington Bosworth, A. M., M. D. Illustrated with nearly 200 engravings and seven full-page chromo-lithographic plates. New York: William Wood & Company. 1896.

The author of this treatise is well known to the medical profession. His name has been conspicuous in literature and he has been recognised as authority on many subjects connected with this department of medicine. His recent treatise on the nose and throat, in two volumes, was reviewed in the Journal, Vol. XXIX., p. 449, and Vol. XXXII., p. 452, at which times our opinion was expressed regarding it in considerable detail.

The present work is mainly a condensation of the one alluded to in the foregoing paragraph, which had been found too voluminous for the use of students. Therefore, the author has thought best to prepare this volume, which is designed especially for the use of both general practitioner and student. In this volume he has eliminated those parts of the former work which were of value only for reference. He has also added some new material, but in all essentials this single volume is the same as the larger edition, with the proviso that Bosworth has brought his observations forward on technical questions, so as to embrace the immediate present. The illustrations, especially in the early chapters, are such as to give the beginner such aid and information as he most desires. The mechanical execution of the book is excellent and the colored plates are graphic. The final section on external surgery of the throat is especially notable as containing advanced thoughts and methods on that subject. We think the author has been wise in reducing his treatise to one volume, which is now ample in size and in wealth of contents to meet the requirements of those whom it is especially intended to serve.

---

A MANUAL OF PHARMACOLOGY AND THERAPEUTICS. By WILLIAM MURRELL, M. D., F. R. C. P., Physician to, and Lecturer on, Pharmacology and Therapeutics at the Westminster Hospital, etc., etc. Revised by Frederick A. Castle, M. D., Member of the committee for revision and publication of the Pharmacopeia of the United States of America, etc., etc. Octavo, pp. 516. New York: William Wood & Co. 1896.

The science of pharmacology has progressed within the last few years so that it may be considered fairly well abreast of its cognate branches, materia medica and therapeutics. We generally find the two latter grouped together in one treatise, but in the work before us we find pharmacology and therapeutics linked together by one author and treated in a single work. The author states that his treatise is an abstract of lectures delivered at the Westminster Hospital, and that it is adapted primarily to the requirements of students preparing for examination. The American editor, Dr. Castle, has revised and rearranged the material, so as to avoid repetitions, economise space and adapt it to the use of Americans. Additions have been made treating of climate and natural mineral waters as a therapeutic aid, and the writings of Dr. Arthur H. Nichols, of Boston, and Geo. E. Walton, of Cincinnati, have been drawn upon liberally in this department. The American editor has made many valuable additions to the text, which he has included in brackets for the sake of identification. One of the features of the work that will appeal to young men is the publication in it of an extensive list of formulæ or prescriptions.

We can commend the book for its general scientific value and for its special adaptation to the requirements of students. It also has many features of interest to practitioners, especially those pertaining to climate and mineral waters, to which we have referred.

---

A PRACTICAL TREATISE ON MATERIA MEDICA AND THERAPEUTICS. By ROBERTS BARTHOLOW, M. A., M. D., LL. D., Professor Emeritus of Materia Medica, General Therapeutics and Hygiene in the Jefferson Medical College of Philadelphia, etc., etc. Octavo, pp. 866. Ninth edition, revised and enlarged. New York: D. Appleton & Co. 1896.

This favorite treatise is before us again for consideration. The eighth edition appeared in 1893, and its early exhaustion indicates a continued demand for the work. We have often told our readers of its excellencies, but do not feel called upon again to state in detail what they consist of. No practitioner of medicine, during the last twenty years, has been unfamiliar with the name of Bartholow or with his writings and teachings on the subject of materia medica and therapeutics. He has been for many years considered one of the ablest therapeutists of the country, and his treatise has been used in most of the colleges since it first appeared.

It is perhaps needless to state that additions and alterations have been made in the present edition, that serve to bring it forward to the period of its issue. It will be found especially rich in its account of synthetic remedies, which are being produced with such rapidity and in such increasing numbers. Bartholow has selected those which in his judgment appear to be best suited to the requirements of physicians, and has described them with scientific detail.

The indexes of the work are especially to be commended. We avail ourselves of this opportunity to express our gratification at the restoration of its distinguished author to health, which at one time seemed almost improbable, and we congratulate him on the resumption of his life-work with all the apparent old-time forcefulness.

---

INTERNATIONAL CLINICS. A Quarterly of Clinical Lectures on Medicine, Neurology, Surgery, Gynecology, Obstetrics, Ophthalmology, Laryngology, Pharyngology, Rhinology, Otology and Dermatology, and specially prepared articles on treatment. By professors and lecturers in the leading medical colleges of the United States, Germany, Austria, France, Great Britain and Canada. Edited by JUDSON DALAND, M. D., (Univ. of Penna.) Philadelphia, Instructor in Clinical Medicine and Lecturer on Physical Diagnosis in the University of Pennsylvania; Assistant Physician to the Hospital of the University of Pennsylvania, etc.; Fellow of the College of Physicians of Philadelphia. J. Mitchell Bruce, M. D., F. R. C. P., London, England, Physician to, and Lecturer on, the Principles and Practice of Medicine at the Charing Cross Hospital. David W. Finlay, M. D., F. R. C. P., Aberdeen, Scotland, Professor of Practice of Medicine in the University of Aberdeen; Physician to, and Lecturer on, Clinical Medicine in the Aberdeen Royal Infirmary, etc. Volume III. Sixth series. 1896. Octavo, pp. xii.—344. Philadelphia: J. B. Lippincott Co. 1896.

Another volume of this interesting series is before us, though like some of its predecessors in the series it contains some material that possesses very little value.

The attempt has been made apparently on the part of a number of young men to creep into this series for the purpose of advertising themselves. Some system of selecting authors should be devised which would prevent any except experienced and recognised teachers from publication in these volumes. On the other hand, most of the material is of an excellent order and deserves high commendation. We desire, however, to enter our protest against such names as "Bartholonitis." Let us get rid of our old personal nomenclature and not manufacture any more.

TRANSACTIONS OF THE MEDICAL SOCIETY OF THE STATE OF PENNSYLVANIA, at its Forty-sixth Annual Session, held at Harrisburg, 1896. Volume XXVII. Published by the Society. Wm. B. Atkinson, M. D., Secretary. Philadelphia : The Edwards & Docker Co., Printers. 1896.

The annual transactions of this state society always furnishes an interesting and valuable contribution to the literature of medicine ; especially is it among the best society transaction volumes constructed within the limits of state lines. Well-published transactions of a working medical society are now more important than ever. Every society should have a complete record of its work, in order that it may be at all times accessible for reference when required. Such a record, too, furnishes an important link in the historical chain of the profession of medicine in the state in which made.

The volume before us contains many interesting papers and indicates a high order of standard among the profession of the Keystone state. A very valuable paper appears on the history of the medical society of the state of Pennsylvania, by Dr. Wm. B. Atkinson, secretary. This contains a brief synopsis of each meeting from the organisation of the society down to the present time.

---

A TEXT-BOOK OF MATERIA MEDICA, THERAPEUTICS AND PHARMACOLOGY. By GEORGE FRANK BUTLER, Ph. G., M. D., Professor of Materia Medica and Clinical Medicine in the College of Physicians and Surgeons, Chicago ; Professor of Materia Medica and Therapeutics, Northwestern University, Women's Medical School ; Attending Physician to Cook County Hospital, etc. Octavo, pp. 858. Philadelphia : W. B. Saunders, 925 Walnut street. 1896.

The wealth of literature on materia medica and its cognate departments, therapeutics and pharmacology, that has been issued during the past year is further increased by the addition of the treatise described in the above title. The object of the work, according to the author, is to supply the student with a practical text-book adapted for permanent reference, as well as for the requirements of the class-room. Butler further states that the arrangements embodying the synthetic classification of drugs, based upon therapeutic affinities, he believes to be the most philosophical and rational, as well as that best calculated to engage interest in the study of the subject.

From a reasonably careful examination of this work we are prepared to affirm that the author has not made unjust claims in regard to the value and importance of the bases that he has adopted in constructing his treatise. It brings before the student a clear, concise and truthful account of each drug, and presents it in a manner calculated to impress the memory. Moreover, it is divested of unnecessary verbiage, and contributes, from the manner of its presentation, to make a subject that is usually dry more attractive and studiable.

The pharmaceutical section of the book has received the special attention of the author, and this increases the importance and value of the treatise. He has enumerated and described only such drugs as experience has proved to be of value and that are accepted in general practice, omitting untried remedies or those of doubtful efficiency. Even official drugs that either have dropped entirely out of use or are next to never employed have been eliminated.

Butler has treated poisonous drugs under two heads,—namely, untoward action, and poisoning, a classification that is both helpful and new. He has adopted the medicinal nomenclature of Foster, and has paid attention to pronunciation and accent, that are both so necessary for the physician of education to observe. He has also taken pains to preserve forms that will be useful in writing prescriptions, carefully observing the Latin genitive, which is so often disregarded by busy practitioners of medicine. We have endeavored to point out a few of the excellencies of this treatise, but there are many others that must be discovered by the owner after he purchases it.

---

Practical Notes on Urinary Analysis. By William B. Canfield, A. M., M. D., Lecturer on Clinical Medicine, University of Maryland, etc. Second, revised, edition. The Physician's Leisure Library. Detroit: George S. Davis. 1896.

We have heretofore spoken of this little work in considerable detail in these colums, and now upon the appearance of the second edition little remains to add excepting to call attention to the fact that certain changes and additions have been made in order to bring it forward to the most modern standard of requirements. In fact, the author says that he has thoroughly revised and corrected the entire edition.

Canfield starts off with a chapter on the general character of the urine which is both interesting and scientific. The second chapter on the normal constituents of urine begins with urea, which is the most important product of decomposition of the albuminous bodies. He gives minute directions for its test that are practical and as simple as possible. Uric acid, oxalic acid and indican are the remaining organic constituents. Next follows the inorganic constituents of the urine, such as chlorides and phosphates; these are briefly though adequately touched upon. The abnormal constituents of the urine are taken up in the third chapter, the first one of which is albumin. This is the one that will attract the attention of the beginner perhaps more than any other substance. It, however, has ceased to have the significance in its relation to kidney diseases that it formerly possessed, and here is where the skill of differentiation is brought to the test.

The next important abnormal constituent considered is sugar. As a transitory condition this, too, may have little significance, but when more lasting it is indicative of that dreaded disease, diabetes melitus. Tests for sugar are admirably given in this treatise. Blood, pus and bile next follow and are briefly and intelligently discoursed upon. Sediment is considered in chapter sixth, and this is really one of the most interesting sections of the work. Detailed directions are given for the microscopical differentiation of sedimentary products. Then comes the urinary diseases in chapter five, reagents, chapter six, and finally the order of analysis in chapter seven, which closes the book and a convenient index is added. This is one of the most helpful brochures on the practical analysis of urine with which we are familiar.

---

THE MEDICAL AND SURGICAL USES OF ELECTRICITY. By A. D. ROCKWELL, A. M., M. D., formerly Professor of Electro-Therapeutics in the New York Post-Graduate Medical School and Hospital, etc. Octavo, pp. xvi.—612. Illustrated with 200 engravings. New York: William Wood & Co. 1896.

This treatise is in reality a successor to, or the ninth edition of, Beard and Rockwell's well-known treatise on the medical and surgical uses of electricity. Dr. Beard has long been dead, and the last edition of the joint work was published in 1892 ; hence it is fitting that a revision should take place. Dr. Rockwell has undertaken the task of rewriting the book, condensing it in places and adding much to it in others, so that it is now brought down to the immediate present and includes among its latest suggestions the surgical application of X-ray photography.

The first part of the book is devoted to the consideration of electro-physics, in which all sorts of electrical apparatus is described in detail. In the next, electro-physiology is considered, and nine chapters are presented in which the effect of electricity on different parts of the body is described. The next section, electro-therapeutics, consisting of thirty-two chapters, presents the treatment of various diseases to which electricity is considered applicable. Finally, in a section of eight chapters, electro-surgery is dealt with, which is one of the most interesting departments in the book. It is in this section that the application of the Roentgen rays is developed.

The work is practically a new one, although it is in another sense well known to the profession through its distinguished author. Every one who is interested in the application of electricity to medicine or surgery will find it inconvenient to do without this book, as it contains much material not to be found elsewhere. It is handsomely printed and bound, and its price is reasonable.

